# Postpartum Ovarian Vein Thrombosis Due to COVID-19 in Pregnancy: A Case Report

**DOI:** 10.7759/cureus.36267

**Published:** 2023-03-16

**Authors:** Ahmet Beyazıt, Oya Soylu Karapınar, Kenan Serdar Dolapçıoğlu, Selen Beyazıt

**Affiliations:** 1 Department of Obstetrics and Gynecology, Mustafa Kemal University, Hatay, TUR; 2 Department of Radiology, Mustafa Kemal University, Hatay, TUR

**Keywords:** postpartum fever, enoxoparin, pregnancy, covid-19, ovarian vein thrombosis

## Abstract

Ovarian vein thrombosis is a clinic condition that is generally seen in the postpartum period and can mimic acute appendicitis with acute abdomen. The incidence of occurrence has increased further in cases predisposing to thrombosis. Coronavirus disease 2019 (COVID-19) during pregnancy causes increased thromboembolic events. Here we examined a case of ovarian vein thrombosis after stopping enoxoparin in a postpartum patient who had COVID-19 during pregnancy.

## Introduction

Ovarian vein thrombosis is a rare entity observed most commonly in the postpartum period. Its incidence rate is 0.5-1 per 1000 vaginal deliveries and 20 per 1000 cesarian deliveries [1]. The classic symptoms are fever and lower abdominal pain that can mimic acute appendicitis. A palpable abdominal mass can also be found [2]. Apart from pregnancy and the postpartum period, it can also be seen in malignancies, pelvic inflammatory events, sepsis and conditions that predispose to thrombophilia (such as rheumatic diseases) [3]. Increased estrogen levels during pregnancy create a tendency to hypercoagulation. Likewise, the increase in the coagulation factors and the decrease in the protein S and antithrombin 3 levels greatly increase the susceptibility to thrombosis [4]. After the birth, spread of bacteria from intrauterine sources especially to the right vein contributes to the pathogenesis [5]. The right ovarian vein is involved in 90% of cases. This is due to longer right ovarian vein lacking competent valves. Also, retrograde flow in the left vein is protective against stasis and ascending infections, therefore there is less risk in terms of thrombosis [6]. Compression of the inferior vena cava and right ovarian vein due to the dextrorotation of the pregnant uterus contributes to this situation. 

Ultrasonography is challenging in diagnosis due to the difficulties in visualizing the ovarian veins because of dense overlying gas. Ultrasound classically demonstrates an enlarged vein with intraluminal echogenic material while Doppler color flow demonstrates reduced or non-existent flow [7]. The most sensitive method in diagnosis is the demonstration of thrombus in contrast-enhanced CT, thus making it the most commonly used diagnostic method [8]. 

Ovarian vein thrombosis can be managed both surgically and medically. Both methods have similar success rates [9]. However, due to the advancements in antibiotics and anticoagulant drugs, management shifted more towards medical treatment. Currently, many treatment protocols recommend broad-acting antibiotics and anticoagulation therapy as treatment modalities [10]. 

Here we present a case of ovarian vein thrombosis that presented 40 days after cesarean delivery.

## Case presentation

A 25-year-old G2P2 patient presented to the emergency department with fewer and right lower abdominal pain. The patient had a history of cesarean delivery 40 days ago, necessitated by previous uterine surgey. Upon questioning, it was learned that the patient didn’t have any complications during and after delivery and she was discharged from the hospital in good health two days after delivery. Patient stated that the fever was present for two days (38 C) and the abdominal pain started one day ago. It was also determined she had tested positive for COVİD-19 in the second trimester of her pregnancy. Upon her D-dimer value being 3390 in the next follow-up, she was started on prophylactic enoxaparin. The patient used enoxaparin throughout her pregnancy and for 30 days after delivery. 

**Figure 1:**
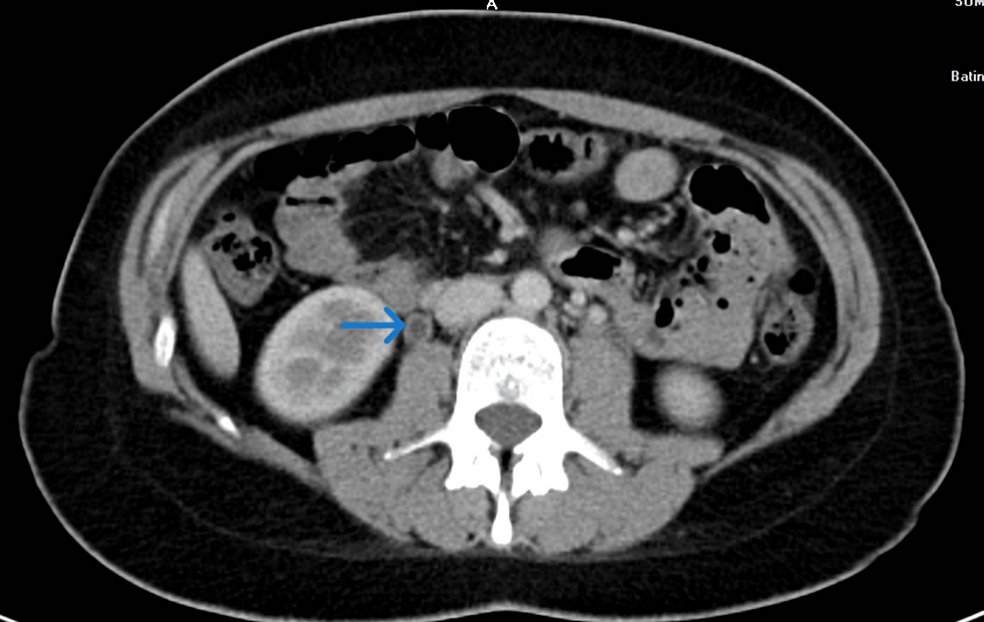
Axial Contrast-Enhanced CT View of Thrombosis

During the first evaluation, the patient’s temperature was 37.6, heart rate 88 beats/minute, blood pressure 110/70 mmHg, and oxygen saturation 98% in room air. Physical examination revealed severe tenderness in the right lower abdomen. There was no abdominal guarding or rebound tenderness. Other body system examinations were unremarkable. Gynecological examination was normal. A mild leukocytosis (11.000) was detected in the laboratory values of the patient. C-reactive protein (CRP) was 12.1 (normal range 0-5); coagulation parameters were normal and D-dimer was 427 ng/ml ( normal range 0-500). Urine analysis detected severe pyuria (+3 in dipstick) and proteinuria (+3 in dipstick). Escherichia coli growth was seen in the urine culture. An abdominal ultrasound was performed to exclude acute appendicitis. After the ultrasound was normal, contrast-enhanced CT was performed and right ovarian vein thrombosis was detected (Figure 1). Subsequent lower extremity Doppler ultrasonography and chest CT did not reveal pulmonary emboli or deep vein thrombosis. The patient was hospitalized; enoxaparin and broad-spectrum antibiotics were started. 

## Discussion

When we look at our case, the patient has risk factors that can be associated with ovarian vein thrombosis. The postpartum period is the highest risk period for ovarian vein thrombosis [11]. However while most of the cases in the literature are seen in two to six days postpartum, the symptoms in our patient appeared 40 days later [12]. We are of the opinion that symptoms did not occur in the most risky period due to the postpartum use of enoxaparin. At the same time, it should be kept in mind that the severe urinary system infection that occurred later in the patient may have contributed to this situation. 

Our case is a patient who had used enoxaparin during pregnancy due to high D-dimer levels thought to be related to COVİD-19, but stopped treatment 10 days ago. It is widely known that COVİD-19 is a condition characterized by high D-dimer levels and an increased tendency for hypercoagulation [13]. When we look at the literature, cases of ovarian vein thrombosis due to COVİD-19 can be seen. Fatizahra et al. published two cases with ovarian vein thrombosis after coronavirus infection, but these two cases were neither pregnant nor postpartum patients [14]. When we look at the covid-related ovarian vein thrombosis cases in pregnancy we see the ovarian vein thrombosis case in a five-week pregnant woman published by Mohammadi [15]. In more published cases we see that there is underlying pelvic surgery or malignancy. 

We think that ovarian vein thrombosis, which occurred in the postpartum period in our patient, is one of the long-term effects of COVID-19. The fact that the patient didn’t experience any thrombotic conditions in her previous pregnancy and during her non-pregnant life and that ovarian vein thrombosis emerged after the discontinuation of anticoagulant treatment supports our thesis.

## Conclusions

Ovarian vein thrombosis is a rare cause of postpartum fever and abdominal pain that can mimic acute abdomen. It should be kept in mind in women presenting with acute abdomen in the postpartum period where thromboembolic complications are extremely common and also in those who have a history of COVID-19. Ovarian veins should be carefully examined during diagnostic tests. 
